# Multimodal Rehabilitative Outcome Measures of Fatigue in Patients with Diabetic Neuropathy

**DOI:** 10.3390/brainsci16030298

**Published:** 2026-03-07

**Authors:** Cira Fundarò, Dibo Mesembe Mosah, Fabio Plano, Roberto Maestri, Stefania Ghilotti, Pierluigi Chimento, Marina Maffoni, Monica Panigazzi, Guido Magistrali, Stefano Bruciamonti, Manuela Ravasio, Chiara Ferretti

**Affiliations:** 1 Neurophysiopathology Unit, Montescano Institute, Istituti Clinici Scientifici Maugeri IRCCS, 27040 Montescano, Italy; cira.fundaro@icsmaugeri.it (C.F.); dibo.mosah@icsmaugeri.it (D.M.M.); pierluigi.chimento@icsmaugeri.it (P.C.); 2 Department of Neuromotor Rehabilitation, Montescano Institute, Istituti Clinici Scientifici Maugeri IRCCS, 27040 Montescano, Italy; fabio.plano@icsmaugeri.it (F.P.); guido.magistrali@icsmaugeri.it (G.M.); stefano.bruciamonti@icsmaugeri.it (S.B.); manuela.ravasio@icsmaugeri.it (M.R.); chiara.ferretti@icsmaugeri.it (C.F.); 3 Department of Biomedical Engineering, Montescano Institute, Istituti Clinici Scientifici Maugeri IRCCS, 27040 Montescano, Italy; roberto.maestri@icsmaugeri.it; 4 Department of General Medicine, Pavia Institute, Istituti Clinici Scientifici Maugeri IRCCS, 27100 Pavia, Italy; stefania.ghilotti@icsmaugeri.it; 5 Psychology Unit, Montescano Institute, Istituti Clinici Scientifici Maugeri IRCCS, 27040 Montescano, Italy; 6 Occupational Physiatry and Ergonomics Unit, Montescano Insitute, Istituti Clinici Scientifici Maugeri IRCCS, 27040 Montescano, Italy; monica.panigazzi@icsmaugeri.it

**Keywords:** type 2 diabetes mellitus, rehabilitation, fatigue, surface electromyography, aerobic training, resistance training, diabetic neuropathy, outcome measures

## Abstract

*Background/Objectives*: Diabetic neuropathy (DN), a common complication of type 2 diabetes mellitus, manifests as peripheral nerve dysfunction with symptoms such as fatigue. Although exercise effectively reduces fatigue in neuropathy patients, precise detection methods are crucial to elucidate the role of rehabilitation. Accordingly, this study aimed to evaluate fatigue in DN patients using a multimodal approach (clinical and instrumental) and to compare the efficacy of aerobic versus resistance training on fatigue parameters. *Methods:* Eligible DN inpatients admitted for rehabilitation at the Neuromotor Rehabilitation Unit of the IRCCS ICS Maugeri Institute of Montescano (PV) were enrolled. Inclusion criteria included age between 65 and 85 years and confirmation via the Michigan Neuropathy Screening Instrument (anamnestic section: ≥7; clinical section: ≥2.5). Patients with confounding orthopedic, neurologic, or unstable cardiopulmonary/diabetic conditions were excluded. Overall, 36 participants were randomized into two groups: 17 underwent aerobic training (treadmill), while 19 received resistance training (elastic bands), both as supplements to a standard rehabilitation program. Assessments at baseline and post-training comprised clinical measures (Borg CR10 scale, Functional Independence Measure (FIM) total and subitems, Six-Minute Walk Test (6MWT), fasting blood glucose) and instrumental evaluations (sEMG of the tibialis anterior muscle to analyze conduction velocity intercept, slope, and changes). *Results:* All patients completed the protocol without dropout or adverse events. Both groups demonstrated significant improvements in FIM scores and post-exercise perceived exertion over time. Instrumental sEMG analysis confirmed a physiological fatigue trend manifested as conduction velocity reduction, yet revealed no significant differences between groups. *Conclusions:* Multimodal assessment provides an effective means to characterize fatigue in DN patients. Both aerobic and resistance modalities enhance functional independence and fatigue perception. Its early identification enables clinicians to tailor rehabilitation strategies to overcome exercise barriers.

## 1. Introduction

Diabetes mellitus (DM) is a chronic disease that can lead to complications such as retinopathy, nephropathy, and neuropathy [1].

Diabetic neuropathy (DN) is a disorder characterized by signs and symptoms of peripheral nerve dysfunction in these patients [2,3]. DN is generally marked by symmetrical sensory disturbances, neuropathic pain, distal weakness, and, in the advanced phase, muscle atrophy [3,4,5,6].

Almost 50% of patients with DM have or will develop neuropathic symptoms [7,8]; however, if subclinical variants (e.g., small fiber neuropathy) [5,8,9] are also considered, two thirds of the population will develop neuropathy [10], occurring more frequently in type 2 DM patients (T2DM) [4,5,6,11]. Distal symmetrical neuropathy is the most common form, accounting for 75% of DN [4].

Among symptoms of DN, fatigue is frequently reported. About 60% of patients with a recent diagnosis of DM experience fatigue [12], with resulting functional consequences [13], muscle impairment [14] and negative effects on quality of life [15,16].

Overall, fatigue has been defined as a subjective symptom or an objective performance decrement [9,10,11,12,13], occurring in numerous other clinical conditions [17,18,19].

More evidence supports the relationship between type 2 diabetes mellitus (T2DM) and fatigue. However, specific recommendations for detecting fatigue in T2DM are currently unavailable [12]; therefore, there is a need to standardize the measurement and diagnostic criteria for fatigue in diabetes [13].

Particularly in DM, fatigue may be associated with hypo- or hyperglycemia; however, it may also be related to psychological factors, such as depression or emotional distress linked to the diabetic condition (e.g., diagnosis or disease management) or to lifestyle factors (e.g., physical inactivity or overweight) [15,20].

In T2DM, muscle fatigability is associated with the presence of DN, and it seems to be related to sensory nerve and peripheral vascular dysfunction too [14].

Regarding the muscular component, fatigue is defined as a decreased capacity to produce maximal force or power output [11]. It is considered a multifaceted phenomenon involving physical and chemical changes within the muscle, distinct from alterations in peripheral nervous system efficiency [11].

In the rehabilitation literature, preliminary evidence generally suggests that exercise therapy is beneficial for DM [21,22]. Specifically regarding DN, physical training appears to provide short-term benefits for neuropathic symptoms, signs, and physical function; however, the quality of evidence supporting these studies remains low [23].

In real-life rehabilitation interventions, a relevant aspect of therapy for DM patients is currently exercise-based physiotherapy, since evidence suggests that aerobic exercise and strength and balance training can improve nerve function, muscle strength, balance, and overall physical performance [24].

Accordingly, the latest guidelines from the American College of Sport Medicine, in collaboration with the American Diabetes Association, recommend at least 150 min per week of moderate-to-intense aerobic physical activity [25]. However, resistance training is also considered beneficial when performed with a load between 50% and 80% of the one-repetition maximum (1RM) [26]. With specific regard to exercise prescriptions in diabetic neuropathy, both resistance and aerobic training have shown clinically relevant benefits in patients with type 2 diabetes. Meta-analytic evidence indicates that resistance training, performed three times per week for up to 12 weeks and targeting major muscle groups with weight machines, free weights, resistance bands, or pneumatic devices (1–4 sets per exercise, 5–20 repetitions, most commonly 8–12), enhances skeletal muscle mass and strength and improves metabolic and inflammatory markers in middle-aged and older adults with T2DM [27]. Similarly, structured aerobic or multicomponent programs conducted more than three times per week, for at least 60 min per session (≥180 min per week), significantly improve aerobic capacity in people with T2DM [28].

Furthermore, regarding fatigue symptoms in neuropathy, research has demonstrated that regular physical exercise is associated with a reduced frequency of fatigue episodes [29]. Specifically, a study conducted on T2DM patients and peripheral neuropathy has demonstrated that aerobic physical exercise improves fatigue symptoms as measured by the Multidimensional Fatigue Inventory [30].

Therefore, the evaluation of fatigue in DN can be crucial in understanding the impact of this symptom and the role of rehabilitation in this context [31].

Several approaches to evaluating fatigue have been employed in rehabilitation and research settings; however, quantifying this symptom remains challenging, particularly in the context of DM, due to the complexity of its clinical interpretation. To the best of our knowledge, among fatigue scales, the most common clinical instruments are the Fatigue Severity Scale (FSS) [32], also available in diabetes; the Fatigue Assessment Scale (FAS) [33]; the Visual Analog Fatigue Scale (VAFS) [34]; and, due to its extensive application in rehabilitation, the Borg scale [35].

In addition to clinical evaluation, instrumental methods are available to assess muscle fatigue during contractions. Among these, in the research field, multichannel surface EMG (sEMG) represents a scientifically established methodology, valid also for DN [36], to explain the myoelectric fatigue phenomenon and to evaluate rehabilitation results [36].

Among sEMG parameters, the muscle fiber conduction velocity is considered particularly reproducible, as it is specific to each muscle in both the upper and lower limbs [11,37,38]. Empirical findings indicate that DPN is associated with modifications in sEMG-derived parameters that reflect impaired muscle activation and endurance. For instance, Park and colleagues [11] reported that type 2 diabetic patients with neuropathy exhibit abnormal sEMG patterns consistent with premature muscle fatigue, while Allen et al. [39] suggested that reduced muscle endurance in DPN may partly result from neuromuscular transmission failure. Complementing these observations, Suda et al. [40] investigated the muscle fiber conduction velocity (MFCV) during different gait phases and demonstrated that individuals with early-stage DPN already show decreased MFCV in lower-limb muscles, particularly in the tibialis anterior and biceps femoris. Interestingly, more advanced neuropathic stages were associated with higher MFCV values in distal muscles, reflecting stage-dependent neuromuscular adaptations. These findings emphasize the relevance of MFCV as a physiologically meaningful and field-applicable indicator of muscle fatigue during functional tasks such as walking, supporting its use in the rehabilitative assessment of neuromuscular performance in DPN. We believe that the choice of appropriate rehabilitative treatment in the management of DN is particularly relevant, and the identification of the fatigue symptom itself is crucial for this purpose.

Since the current guidelines on the management of DM do not specify which type of rehabilitation is recommended for this condition [34], we decided to use treadmill training for aerobic exercise, while elastic resistance bands were used for resistance training [41].

Specifically, the aims of this study were (1) to assess the fatigue phenomenon in the DN population without evidence of autonomic neuropathy using a multimodal perspective (clinical and instrumental) and (2) to compare aerobic and resistance training through multimodal outcome measures, with a specific focus on fatigue parameters. In more detail, primary outcomes included the Borg CR10 scale [35] and muscle fiber conduction velocity changes assessed via sEMG. Secondary outcomes addressed related factors: motor capacity (Six-Minute Walk Test, 6MWT) [42], clinical status (fasting blood glucose), and functional independence (Functional Independence Measure (FIM scale), total and subscores) [43].

## 2. Materials and Methods

### 2.1. Data Collection

Consecutive inpatients admitted for rehabilitation in the Neuromotor Rehabilitation Unit of the IRCCS ICS Maugeri Institute of Montescano (PV) with a diagnosis of T2DM [1,2] were assessed for this study by submitting them to clinical and instrumental evaluations to confirm the diagnosis of diabetic peripheral neuropathy (DN). Participants were recruited from 1 April 2018 to 31 October 2019 (18 months).

A multidisciplinary board (i.e., neurophysiopathologist, neurophysiopatholy technician, physiatrist, neurorehabilitation therapist, and diabetologist) was involved throughout the study, from design to training and the evaluation of the results.

### 2.2. Study Criteria

The following criteria were applied for patients’ recruitment.

Inclusion criteria included a clinical [44] and electroneurography (ENG) diagnosis [45] of diabetic polyneuropathy [8,46,47]; age between 65 and 85 years; a score of ≥7 on the anamnestic section and a score of ≥2.5 on the clinical section of the Michigan Neuropathy Screening Instrument (MNSI) [48]; and the provision of informed consent.

The following exclusion criteria were applied to ensure the feasibility, tolerability, and safety of the trial, as well as accurate instrumental recording and data evaluation: orthostatic hypotension; other autonomic dysfunctions (e.g., cardiovascular or gastrointestinal autonomic neuropathy); recent modifications to antidiabetic therapy (within the last week); recent participation in rehabilitative training programs (within the last 2 months); recent fractures of the lower limbs, forefoot amputations, skin sores of the legs or feet, recent joint replacement surgery, severe osteoarthritis, or other musculoskeletal conditions impairing treadmill/exercise performance; unstable blood glucose levels (i.e., unpredictable hypoglycemic episodes), unstable cardiopulmonary disorders, lumbar and/or radiculopathy, cognitive impairment, or other neurologic disorders (e.g., Parkinson’s disease, neuromuscular junction disorders, demyelinating disease, stroke, etc.).

Moreover, participants were withdrawn from the study in the case of relevant intercurrent organic events (e.g., infections, respiratory decompensation, glycemic instability), safety concerns related to exercise training, changes in diabetes therapy during the intervention period, or voluntary withdrawal of informed consent. No participants met these criteria during the study.

### 2.3. Study Design and Intervention Training

This was a randomized interventional study. Patients were randomly divided into two groups.

**Figure 1:**
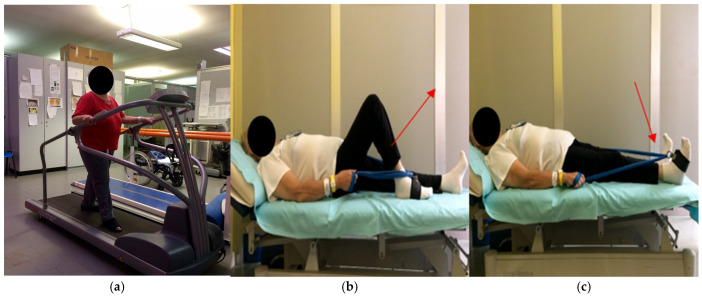
(**a**–**c**) Patient performing exercises. Note: In (**a**), the patient participated in aerobic training with a treadmill; (**b**,**c**) illustrate hip extension resistance training using Thera-Band^®^ elastic bands, targeting the hamstring and gluteal muscles. Arrows indicate the movement direction: from a flexed hip/knee position through concentric extension to full straightening, promoting strengthening via elastic tension (**b**); subsequently, the extended limb is pushed toward the bed plane (**c**).

*Group 1*: Patients performed *aerobic training on a treadmill*, supervised by a physiotherapist (Figure 1a). The parameters of this training were as follows: 5 days per week for 4 weeks (frequency), reserve heart rate [49] (intensity), 30 min session (duration). The treadmill speed and gradient were adjusted to maintain the training intensity within the desired range; the treadmill speed was further modulated to ensure walking rather than running. Heart rate and oxygen saturation were monitored throughout the training session using a finger pulse oximeter.

The intensity of aerobic exercise (40–60% of reserve heart rate) was determined using the Karvonen formula [49]. This method considers individual fitness levels by incorporating both maximal and resting heart rates. The heart rate reserve (HRR) was calculated as the difference between the maximal heart rate (220—age) and resting heart rate, and the target heart rate was obtained by multiplying the HRR by the desired training intensity (e.g., 0.4–0.6) and adding back the resting heart rate. Intensity was monitored in real time using a fingertip pulse oximeter and supervised by a licensed physiotherapist. Accordingly, adjustments were made during the aerobic exercise sessions to align the absolute workload with the target relative intensity.

*Group 2*: Patients performed *resistance training with Thera-Band^®^ elastic bands* [50]. The parameters of this training were as follows: 5 days per week for 4 weeks (frequency), with 3 sets of 20 repetitions of each prescribed exercise for both lower limbs for a total 30 min session (duration). Intensity was set at 50% of the estimated 1RM [26]. Due to the non-linear resistance profile of elastic bands, the 1RM was estimated individually for each exercise and participant. Specifically, the 1RM is defined as the greatest load that a person can lift for a single repetition with the correct technique in a given exercise; it is typically determined by progressively increasing the load until the maximum weight that can be lifted once is identified.

The use of elastic resistance during exercise is intended to increase strength and the range of motion in the body region on which the exercise is performed. The resistance offered by each elastic band is proportional to the percentage of its elongation, i.e., a greater range of movement generates higher resistance from the band. The Thera-Band^®^ resistance levels at 100% elongation (length doubled from resting state) were as follows: yellow = 1.3 kg, red = 1.7 kg, green = 2.1 kg, blue = 2.6 kg [49].

Two exercises were selected to engage all lower-limb muscle chains involved in walking. Specifically, one exercise focused on strengthening the anterior muscle chain, while the second strengthened the posterior muscle chain (Figure 1b,c). This tool was chosen for its simplicity, safety, and low cost.

To standardize the training sample, yellow, red, green, and blue bands were used, representing increasing levels of resistance (from lowest to highest, respectively). Specifically, the elastic resistance bands were progressively advanced (yellow → red → green → blue) according to each patient’s individual progress and tolerance throughout the intervention, as described in the Thera-Band progressive resistance training protocols suitable for older adults with diabetes [51]. The bands were adjusted uniformly for all group 2 patients throughout the training period.

The parameters of aerobic and strength training are informed by established meta-analyses demonstrating their efficacy in improving neuropathic symptoms and physical function in DPN patients [27,28].

### 2.4. Conventional Training

In addition to the specific training sessions described above, both groups of patients underwent the standard physiotherapeutic protocol for patients with neuropathy used in the IRCCS ICS Maugeri of Montescano Institute [52]. This protocol included the passive mobilization of the limbs, stretching of the main muscle chains of the lower limbs, neuromuscular facilitations, muscle strengthening of the trunk and upper-limb muscles with manual resistance, and proprioceptive balance training on stable/unstable surfaces. This treatment program was delivered 5 days per week for 4 weeks for a 70 min session per day, and the exercises were performed in a daily rotation.

### 2.5. Safety

For the entire duration of the training sessions, the heart rate and oxygen blood saturation were collected. Moreover, blood glucose levels were monitored before, during (if clinically indicated), and after each training session using a glucometer to detect and prevent hypoglycemic or hyperglycemic fluctuations induced by exercise. Additionally, potential adverse events such as exercise-induced pain, muscle soreness, or sleep disturbances were systematically recorded at each session. No adverse events, hypoglycemic episodes, or safety concerns were reported throughout the study.

### 2.6. Randomization Procedure

Random allocation into the two study groups was performed using dedicated computer-generated random sequence software implementing a centralized block randomization algorithm (block size = 15) to ensure a quantitative balance across groups. Randomization was managed centrally by the Bioengineering Department. The allocation sequence was kept concealed until allocation, so that the enrolling staff was informed of each patient’s assigned treatment only at the beginning of training. Allocation was concealed using sequentially numbered, opaque, sealed envelopes to prevent selection bias.

**Figure 2:**
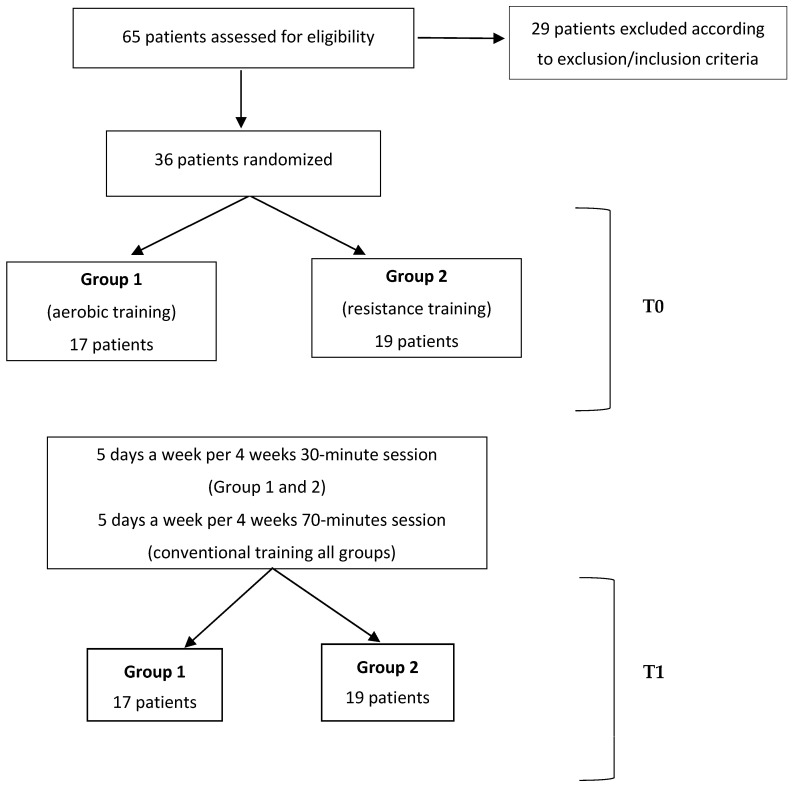
Flow chart of the patients’ screening, recruitment, and training.

Figure 2 summarizes the patients’ screening, recruitment, and training.

### 2.7. Outcome Measures

The clinical evaluation was performed at T0 (i.e., the day before starting training); outcome measures were collected at T0 and at T1 (i.e., the day after finishing the training). Given the clinical heterogeneity of the DN population, the Michigan Neuropathy Screening Instrument (MNSI) was administered to all participants at baseline to ensure sample homogeneity [48]. Individuals scoring less than 7 points on the questionnaire and less than 2.5 points on the physical examination were excluded. The diagnosis of neuropathy was subsequently confirmed by electroneurography (ENG).

Multidimensional outcome measures assessed motor, physical, functional, and instrumental performance; perceived exertion (Borg CR10) [53]; the independence level (FIM) [43]; and muscle fatigue (sEMG). Blood glucose levels were assessed before and after training sessions.

The outcome measures are described below.

#### 2.7.1. Primary Outcomes

− The Borg Category Ratio Scale 0–10 (Borg CR10) [53] is a scale used to evaluate a patient’s perception of effort and exertion, breathlessness, and fatigue during physical activity. It is an outcome measure for exercise modulation. The scale ranges from 0 to 10 points, where 0 is no exertion at all and 10 is the maximum exertion. Borg CR10 ratings were assessed pre- vs. post-session at T0 and at T1. These measurements captured acute within-session fatigue changes at the beginning and end of the intervention period. Accordingly, Borg ratings are reported in the manuscript as Borg CR10 pre-treatment and Borg CR10 post-treatment, in both T0 and T1.
− The FIM^®^ scale [43] is a questionnaire designed to measure the degree of a patient’s disability. The items cover 18 activities of daily living, of which 13 relate to motor and sphincter functions and 5 to cognitive functions. Each of the 18 activities is scored on a scale from 1 (complete dependence on others) to 7 (complete independence). The FIM total score serves as an indicator of the effectiveness of rehabilitation and hospitalization. It is possible to have a motor score and a cognitive score too.

#### 2.7.2. Secondary Outcomes

##### Clinical Measures

− The MNSI [48] is a specific assessment tool for patients with peripheral neuropathy, divided into two sections. The first consists of a patient history questionnaire addressing key symptoms; the second involves a physical examination performed by a physician. The history section includes 15 self-administered questions (maximum 15 points). “Yes” scores 1 point for all questions except n. 7 and n. 13, where “No” scores 1 point. The physical examination section must be performed with the patient’s feet warm (>30 °C). Foot inspection is used to detect excessively dry skin, callused deformities, fissures, ulcers, or structural abnormalities (amputation). Vibratory sensitivity was tested using a 128 Hz tuning fork placed on the large toe, with the patient’s eyes closed. Achilles tendon reflexes were assessed with the patient seated and relaxed. The monofilament test was applied to the large toe with a perpendicular force of 10 g, with the patient’s eyes closed. The maneuver is repeated 10 times: 8 correct responses are considered normal; 1 to 7 correct responses indicate reduced sensitivity; no responses indicate absent sensitivity.
− The 6MWT [42] is used to measure the maximum distance that patients can walk in 6 min. It is performed by walking back and forth between two cones at 30 m. Patients are instructed to walk as much as possible, for six minutes, and walking aids such as walkers or canes are allowed if needed. The test is easy to perform, well tolerated, and safe for the patient. Heart rate (HR) and oxygen saturation (SpO2) were recorded at baseline (T0) to characterize the study population and were monitored during training sessions to ensure patient safety.

At baseline, we collected also sociodemographic (e.g., gender) and clinical variables, which included age (years), BMI (kg/m^2^), gender, smoking status, diabetes complications (e.g., retinopathy, nephropathy), blood glucose (mg/dL), heart rate (HR pre-treatment, bpm), and SpO2 pre-treatment (%).

##### Instrumental: sEMG Evaluation

The myoelectric localized phenomenon of muscle fatigue was analyzed using multi-channel sEMG [54,55]. The following equipment was used: (a) sensors for isometric torque measurements of the ankle to measure strength MISO1 (Department of Electronics and Telecommunications, LISiN, Laboratory of Engineering of Neuromuscular System, Polytechnic University of Turin, Turin, Italy); (b) a visual feedback system, indicating the level of contraction to be sustained; (c) a modular mechanical brace to hold and immobilize the lower limb, incorporating two torque transducers independently connected to the amplifier; (d) a 16-channel amplifier; (e) a flexible adhesive linear array of 16 electrodes (i.e., 10 mm inter-electrode distance in a single differential configuration); (f) a ground electrode; (g) a TV monitor to display the EMG signal; (h) a Vectra Celeron PC (Hewlett-Packard, Palo Alto, CA, USA); (i) the Microstar software (version 32.0, Microstar Laboratories, Bellevue, WA, USA) program to acquire signals (EMGACQ/32), analyzed offline via MATLAB (MathWorks, Natick, MA, USA). The data were acquired, stored on a hard disk, and elaborated offline with a dedicated program (EMGACQ/32) [55].

**Figure 3:**
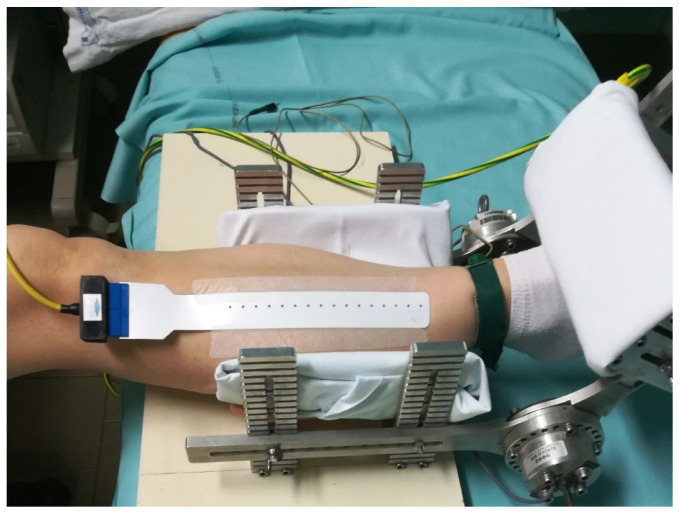
sEMG study setting. Note: In this representation, the patient’s right leg is placed inside the two-mechanic torque of measure; the electrode array and the ground electrode are, respectively, visible on the belly of the right tibial anterior and at the ankle.

sEMG signals were recorded with the foot positioned comfortably inside a brace at a 120° angle to the leg and fixed to ensure conditions for isometric measurements during voluntary dorsiflexion contractions. In this way, the ankle was in the same axis as the brace. Two independent torque transducers were incorporated into the axis of the brace to measure torque, expressed in Nm, which was then summed and displayed through a visual feedback system to guide the subject on the level of torque produced (Figure 3). The skin was cleaned by slightly abrading it with an abrasive gel before positioning the array [55].

The optimal position and orientation of the array were determined at moderate contraction levels of the tibialis anterior [56,57] through visual inspection of the signal by observing clear motor unit action potentials with similar propagation in two directions from the neuromuscular junction to the tendons [55,56,57,58,59].

The ground electrode was placed at the ankle.

The room temperature was monitored and maintained within a range of 30.5 +/− 0.5 C for all subjects and recordings [55].

**Figure 4:**
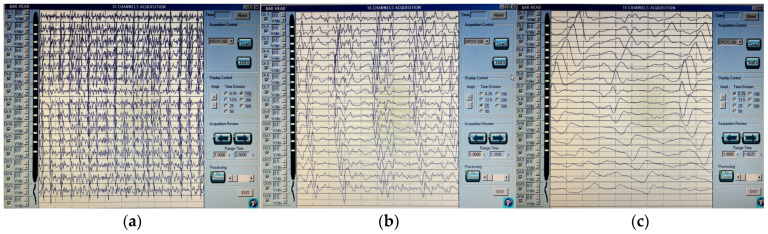
Recordings from sEMG. Note: On the ordinate axis, there is the representation of a linear array of 16 electrodes, and, on the abscissa axis, there is the motor unit action potential propagation. Motor unit potentials with similar propagation in two directions from the neuromuscular junction to the tendons are shown. The motor unit action potential has the same amplitude (0.5 mV/div), while the time division is represented in this figure across (**a**–**c**), where (**a**)—time division 100 ms/div, (**b**)—time division 25 ms/div, and (**c**)—time division 6.5 ms/div.

In this study, the variable used to describe the sEMG signal was the conduction velocity (CV). The variation in this parameter during sustained isometric contraction over time constitutes one of the myoelectric localized manifestations of muscle fatigue [37,51,52,53,54,55,56,57,58,59,60,61,62,63]. Typically, this variable shows an almost linear decrease over time during fatiguing contractions [37,56,57,62]. The data for the analysis were obtained from the channels with the best signal resolution; an example is shown in Figure 4. The rate of change was defined as the slope of the regression line, expressed as a percentage, while the initial value of CV was defined as the intercept, expressed in m/s [55,56,61].

It should be highlighted that sEMG quantifies objective muscle fatigue via physiological alterations elicited during a standardized exercise ramp, reflecting neuromuscular mechanisms that are physiologically induced and necessary for adaptation. Conversely, the Borg [53] and FIM [43] scales evaluate perceived fatigue during training sessions, focusing on the subjective experience rather than peripheral mechanisms. This complementary approach distinguishes neuromuscular fatigue (unavoidable during exercise) from perceptual fatigue (targeted by rehabilitation to enhance tolerance).

### 2.8. Protocol for Recording of Myoelectric Localized Manifestations of Fatigue

Patients were asked to perform three brief maximal voluntary contractions (MVCs) of the right tibialis anterior muscle for 3 s each, separated by a 2 min rest period. The MVC with the highest value was chosen as the reference (100%) for the subsequent voluntary contractions (20% and 80%) [19,55].

**Figure 5:**
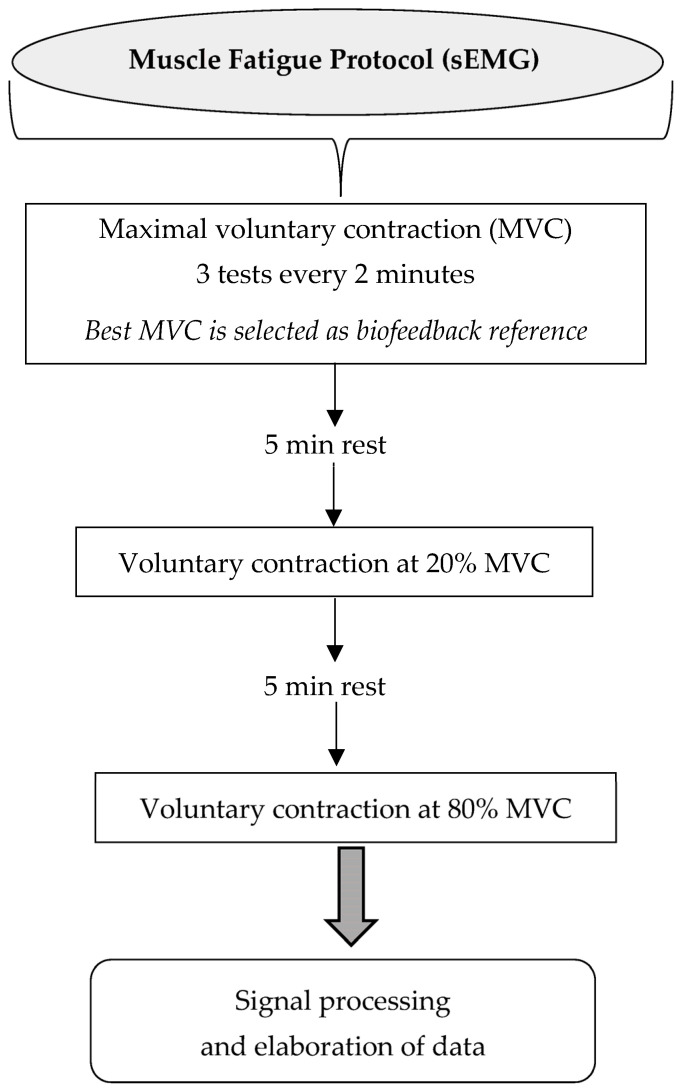
Flow chart of the muscle fatigue recording protocol.

Patients were asked to perform voluntary contractions of the tested muscle at two different levels of MVC (i.e., 20% for 60 s and 80% for 30 s). Visual feedback was used to help the subject to obtain and maintain the requested contraction level. In addition, verbal encouragement was given to optimize patients’ performance. Each contraction was followed by a rest period according to the intensity of the contraction executed (5 min rest at 20% MVC; 10 min rest at 80% MVC) (see flow chart in Figure 5) [55].

The signals were recorded with an array of 16 electrodes/channels: Ch1-Ch15 in single differential mode (SD), while Ch16 was used to measure the force produced during the contraction. Sampling frequency: 2048 Hz. Y div: 1 V/div. X div: 6.5 ms. The epoch length was 0.5 s. The signals were stored on a PC, and signal processing was performed using MATLAB and tabulated in an Excel file [55].

### 2.9. Sample Size

The sample size was calculated considering the Borg CR10 as the primary outcome of the study. Based on previously published studies on the reliability of the Borg scale in test–retest protocols [64], we obtained an estimate of the value of the standard deviation of the differences equal to 0.4 for low-intensity exercise. Subsequently, we computed the minimum sample size required to detect a difference of at least 0.4 (approximately 20% of the mean baseline value) in the change (discharge–baseline) in the Borg scale between the treadmill training and the Thera-Band^®^ elastic groups, with 80% power and a two-tailed type I error rate of 0.05. The resulting sample size was 34 patients (n. 17 in the treadmill group, n. 17 in the Thera-Band^®^ elastic group). Calculations were performed using the proc power procedure of the SAS statistical package (SAS/STAT, release 9.4, SAS Institute Inc., Cary, NC, USA).

### 2.10. Statistical Methods

The normality of all continuous variables was assessed using the Shapiro–Wilk test, supported by visual inspection of the data distribution. Since several variables violated the normality assumption, the results are summarized as medians (lower quartile, upper quartile) for continuous variables and as numbers (percentages) for categorical variables. Between-group comparisons (treadmill vs. Thera-Band^®^ elastic training) were performed using the Mann–Whitney U test for continuous variables and the chi-squared or Fisher’s exact test for categorical variables. Associations between variables were evaluated using Spearman’s correlation coefficients.

To test the hypothesis that the type of treatment could differentially affect rehabilitation outcomes, we applied a generalized linear mixed model (GLMM) to account for the longitudinal design and within-subject correlation of repeated measurements. The model included group (treadmill training and Thera-Band^®^ elastics), time (T0 and T1), and their interaction (group × time) as fixed effects, with the baseline value (T0) of each outcome included as an adjustment covariate. A subject-specific random intercept was included to account for inter-individual variability and the correlation among repeated observations. Results from the GLMM are reported as model-based estimated means and 95% confidence intervals, back-transformed to the original scale, together with the corresponding *p* values for time, group, and the group × time interaction.

All statistical tests were two-tailed, and significance was set at *p* < 0.05. Analyses were performed using the SAS/STAT statistical package, version 9.4 (SAS Institute Inc., Cary, NC, USA).

### 2.11. Ethical Statement

Enrollment proceeded only after written informed consent was obtained. Additionally, participants were informed of their right to withdraw at any time without penalty, and no financial compensation was offered. The study was conducted in accordance with the Declaration of Helsinki’s ethical principles for medical research involving human subjects [65].

This study received approval from the ICS Maugeri Ethics Committee (n. 2185 del 6.3.2018) and was registered on the clinicaltrials.gov register with reference NCT04467255.

The patients’ clinical data were collected in an anonymized database.

## 3. Results

All enrolled patients underwent the training program provided by the study, as well as the clinical and instrumental evaluations scheduled at T0 and T1, except for one patient who did not undergo the T1 sEMG registration due to discharge and one patient who failed to perform the sEMG evaluation at both T0 and T1. Specifically, Borg CR10 ratings were assessed pre- vs. post-session at T0 and at T1. No dropouts or adverse events were reported. Figure 4 illustrates the details of the patients screened, excluded, and recruited.

At T0, no significant between-group differences were observed in the continuous outcome variables, except for the FIM cognitive score (*p* = 0.015, slightly higher in the elastic band group) and FIM motor score (*p* = 0.022, lower in the elastic band group) and, regarding clinical fatigue detection, the Borg CR10 pre-treatment (*p* = 0.039, higher in the elastic band group) (Table 1).

At T0, BMI was significantly associated with the 6MWT (positive relationship) and with the FIM cognitive score (only negative relationship) (Table 2).

Moreover, at T0, no significant differences were found in the continuous clinical variables between subjects with a disease duration <10 years versus >10 years (all *p* > 0.15) (Table 3). In addition, at T0, no significant between-group differences were observed in dichotomous variables (gender, disease duration, insulin treatment, hypertension, smoking, alcohol consumption, dyslipidemia, presence of retinopathy, and presence of nephropathy) (Table 4).

Concerning the instrumental evaluation of fatigue through sEMG, the comparison of the muscle fiber conduction velocity (i.e., CV intercept value 20% and 80% maximal voluntary contraction—MVC) at pre-training showed population homogeneity. Furthermore, the muscle fiber conduction velocity of the tibialis anterior muscle in each patient and group was compatible with normative values for this muscle [57].

Pre- and post-training sEMG recordings confirmed the expected fatigue trend [54,61], namely the reduction in the muscle fiber conduction velocity (CV slope parameter) [54] at both 20% MVC and 80% MVC, with no post-training differences between groups.

Table 5 reports descriptive statistics for the considered outcome measures, for both groups, at T0 and T1. Regarding the efficacy of the two interventions (Table 6), overall, the generalized linear mixed model analysis showed a significant effect of time for many clinical and functional outcomes, indicating an improvement from baseline (T0) to post-intervention (T1) in both treatment groups; significant reductions over time were observed in metabolic and perceived exertion measures, including blood sugar (*p* for time = 0.017), Borg CR10 pre-treatment (*p* = 0.003), and Borg CR10 post-treatment (*p* = 0.01). In addition, functional capacity significantly increased over time: the 6MWT improved markedly in both groups (*p* < 0.0001). Similarly, significant improvements were found in rehabilitation outcomes: the FIM motor score (*p* < 0.0001) and the FIM total score (*p* < 0.0001). These findings suggest that both interventions were associated with meaningful improvements over the study period. No statistically significant main effect of group was detected for any of the outcomes (*p* for group ranging from 0.10 to 0.90). The group × time interaction was not significant for most variables: this suggests that the magnitude of improvement from T0 to T1 did not differ significantly between the two intervention groups, supporting the conclusion that both treatments had similar longitudinal effects. A significant interaction was observed only for the CV intercept value 20% (*p* for interaction = 0.01), indicating a different temporal pattern between groups: that in the treadmill group increased from 3.99 to 4.62, while that in the elastic band group decreased from 4.14 to 3.93. Given that this was the only significant interaction among multiple outcomes, this finding should be interpreted cautiously and may warrant further investigation. For all outcomes, the baseline values were highly significant predictors (*p* baseline < 0.0001 in most models), confirming the importance of adjusting for baseline status when assessing treatment effects.

## 4. Discussion

### 4.1. Clinical Context and Safety in Rehabilitation

DN is a common complication of T2DM, characterized by sensory loss, motor impairment, fatigue, and neuropathic pain. These symptoms significantly increase fall risks and reduce mobility and quality of life [3,16,66]. Moreover, DN is often correlated with muscle fatigue in patients’ experience [11].

Pharmacological treatments often provide limited relief and do not halt disease progression [66], highlighting the need for effective non-pharmacological interventions [25,67,68]. In general, for all individuals with DM, baseline physical activity and time spent on sedentary behavior should be evaluated, and those who do not meet activity guidelines should be encouraged to increase their physical activity above the baseline [21]. While the optimal type and intensity of exercise remain under investigation [29], current findings support the integration of structured exercise programs into standard care for individuals with diabetes neuropathy [21].

It is known that there is a potential regenerative effect on nerve fibers in T2DM patients receiving rehabilitation, compared with the loss of nerve fibers in those who only follow the standard of care [69]. The plastic characteristics of the peripheral nervous tissue as a response to physical exercise show a variation in the intraepidermal fiber density, probably as a consequence of an increase of neurotrophin levels following physical exercise—in particular, brain-derived neurotrophic factor (BDNF) and nerve growth factor (NGF) [70,71].

Nevertheless, despite the well-founded recommendation to engage in regular exercise, patients with DM—especially those with complications—must be constantly monitored to ensure safety during training (Kluding). In the absence of specific guidelines [72], rigorous patient screening (e.g., glycemic balance evaluation, vascular risk factors) [73], clinical monitoring during training (e.g., recording of heart rate and oxygen saturation), and the involvement of a diabetologist in the rehabilitation team are crucial to ensure safety. This approach was successfully employed in our study, where we observed no adverse events or dropouts.

Moreover, regarding the design of tailored training for patients with DM, it appears necessary to consider specific predictors of safety and efficacy, such as frailty indicators. For instance, in our sample, characterized by overall good glycemic balance and DN as the main complication, BMI was significantly related to motor (6MWT) and cognitive performance (FIM cognitive score, negative correlation); this suggests that nutritional status may directly influence physical performance and negatively impact cognitive frailty [74].

At the same time, there is little evidence regarding the impact of peripheral neuropathy on frailty; however, we suggest that DN and fatigue may be correlated with the early onset of functional decline in this population [75].

### 4.2. Methodological Considerations and Patient Selection

The potential heterogeneity of patients with DM raises other methodological considerations that deserve attention. Specifically, we believe that considering only the diagnosis of T2DM and the presence of neuropathy might not have been sufficient to identify two homogeneous groups. Therefore, the use of the MNSI allowed us to standardize patient selection; consequently, the interpretation of the rehabilitation results was more unambiguous [29]. In this regard, in our study, at baseline, no significant differences were found in disease duration between groups. Similarly, no significant between-group differences were observed for any dichotomous variables (i.e., gender, insulin treatment, hypertension, smoking, alcohol consumption, dyslipidemia, presence of retinopathy, and presence of nephropathy). Moreover, the use of the MNSI tool [48] for patient selection, combined with ENG preliminarily performed to confirm the diagnosis of DN, was beneficial in ensuring population homogeneity [8,46,47,66]. Additionally, in terms of feasibility, the diagnostic instruments employed in the study are also easy to realize and to extend to a larger sample.

Therefore, in our opinion, the MNSI as a screening test for DN and the execution of electrophysiological studies may also be proposed in clinical practice to identify the initial stage of DN for the purpose of applying early rehabilitation interventions.

### 4.3. Assessment of Fatigue: Clinical vs. Instrumental Findings

Regarding DN symptoms, the detection of clinical fatigue may be complex. In our sample, using the Borg CR10 scale, the Borg CR10 pre-treatment indicator showed a low level of fatigue overall, which was slightly higher in the elastic band group.

In addition, for detecting fatigue, the integration of the sEMG methodology may be supportive to register this phenomenon from a muscular point of view [11,54,61].

It is known that fatigue is a relevant symptom, and about 50% of the clinically asymptomatic subjects with DM still showed sensory involvement and muscle fiber CV abnormalities, despite standard needle EMG and force [70,76,77].

Furthermore, concerning the instrumental evaluation of fatigue through sEMG, the pre-training comparison of the muscle fiber conduction velocity (CV intercept value 20% and 80% MVC) confirmed population homogeneity. The conduction velocity of the tibialis anterior muscle [57] in each patient and group was also consistent with normative values for this muscle, indicating the good reproducibility of the method used in this study [37,56].

Moreover, in our study, pre- and post-training sEMG recordings showed the expected fatigue trend, as expressed by the reduction in the muscle fiber slope CV at both 20% MVC and 80% [54,61].

The multimodal (clinical and instrumental) evaluation of fatigue, proposed in this study, may provide different and multifaceted information. Indeed, while our population reported generally low levels of perceived pre-training fatigue (Borg CR10), signs of fatigue were nonetheless present and detectable at the muscular level (sEMG).

Although glycemic control seems to play an important role in muscle fatigue mechanisms in subjects with DN, our patients—being sufficiently stable—perceived little fatigue, which was instead detected instrumentally via sEMG [11].

In addition, it is unclear whether long-term T2DM complications play a role in muscle fatigue, but, among these, the presence of DN seems to be significant [11], especially for quality of life [11]. From an interpretative point of view, muscle fatigue in DN appears to be mediated predominantly by sensory nerve and peripheral vascular dysfunction [14,78].

As final clinical remarks, detecting muscle fatigue allows for better management of exercise-induced fatigue, which is a significant barrier [78,79] to physical activity in people with T2DM, exacerbated by impaired tissue healing and chronic inflammation [80].

Moreover, there is generally great variability in perceived exertion in subjects affected by T2DM [81], as well as regarding physical training [82].

### 4.4. Efficacy of Rehabilitation Interventions

Exercise training represents a cornerstone of DPN management, as it alleviates neuromuscular dysfunction—including deficits in muscle strength, power, and fatigue resistance—through neural and muscular adaptations. Specifically, aerobic training enhances neural structure and function, while resistance training improves muscle performance and reduces neuropathic pain, with combined protocols restoring sensory nerve integrity and overall function in this population. These benefits provide the rationale for our combined aerobic and resistance training protocol, which targets both peripheral neuromuscular impairments and functional outcomes in DPN rehabilitation [83].

Concerning the efficacy of the two interventions studied, a time effect was observed in both patients undergoing treadmill training and elastic band training for the Borg CR10 pre-treatment and 6MWT. Specifically, our sample demonstrated improvements in detected muscle fatigue [84] and motor performance (6MWT) [42] over time, regardless of the training type. In addition, the Borg CR10 post-treatment scores decreased in both groups, further demonstrating the influence of physical exercise on perceived exertion.

What is known is that Thera-Band^®^ elastic-assisted training may alleviate dysfunction in DM patients with fatigue, and it appears promising for clinical application and research interpretations about peripheral neuroplasticity [34,70,78], determining, according to some authors, a form of “muscle conditioning” [85]. However, heavier resistance training with free weights or weight machines may improve glycemia and strength [86]. Overall, resistance training of any intensity is recommended to improve strength, balance, and ability in order to engage in activities of daily living throughout the life span [87].

In addition, aerobic training also appears to have a positive effect on fatigue in T2DM patients, likely due to its action on systemic inflammation [88].

Concerning functional abilities and consequently quality of life, in our study, FIM parameters—especially the FIM motor score and FIM total score—improved in both groups. In a pilot study conducted on patients with DN, it has also been demonstrated that aerobic exercise can reduce neuropathic pain [89,90] and influence daily activities more extensively [15,91].

Moreover, exercise plays a key role in enhancing nerve function, reducing the risk of DN, and benefiting neuropathic symptoms and functional capacity; however, its effects on glycemic control remain inconclusive, showing significant variability in study outcomes [11,92,93]. Similarly, in our study, the blood sugar level decreased after treatment in both training groups. Moreover, regarding the instrumental evaluation through sEMG, we did not observe a strong correlation between the type of training, blood sugar level, and sEMG parameters, differing from other experiences [11], likely due to the limited sample size.

While DN is recognized as the main DM complication driving muscle fatigue in T2DM [12,15], our study found no significant clinical differences between groups regarding DM characteristics (i.e., duration of DM and glycemic metabolism), likely due to the well-balanced glycemic status of our sample [11]. Nevertheless, as previously commented, sEMG proved to be a useful outcome for detecting muscle fatigue in patients with DN [11], revealing more specific points for reflection on the muscle fatigue phenomenon and training efficacy too.

### 4.5. Myoelectric Localized Muscle Fatigue from a Diabetic Neuropathy Perspective

In terms of sEMG, regarding the CV intercept value 20%, we observed a significant difference based on the disease duration (<10 years versus >10 years) [76]. Specifically, patients with a history of diabetes longer than 10 years exhibited higher conduction velocities compared to those with a shorter duration. This finding is consistent with Nyholm et al. [94], suggesting a potential shift towards faster type II muscle fibers in patients with a longer disease duration.

This result is in line with the scientific literature, reporting that the muscles have different degrees of responsiveness to the effects of DM and show a modification in the conduction velocity as neuropathy progresses [38].

Specifically, for the tibialis anterior muscle, it is known that fiber conduction velocities estimated with sEMG under physiological conditions are influenced by the muscle’s distal anatomical localization and its fiber type composition, namely the prevalence of type I fibers [95,96,97,98].

During isometric exercise at relatively low contraction levels in the tibial anterior muscle, a condition reproduced in our DM sample, a decrease in the CV intercept value 20% MCV was observed, without significative differences between groups. This may be consequent to the general initial motor unit recruitment of predominant fatiguing type I motor units, followed by the recruitment of fresh type II motor units [99,100].

These observations in patients with DM may be due to neuropathy and, consequently, to the impaired activation of motor unit discharge [101,102]. It is likely that T2DM patients continue to recruit a limited number of the same motor units during sustained contractions at low force levels [36,100]. Ultimately, this results in decreased endurance during isometric fatigue and neuromuscular dysfunction [14].

### 4.6. Study Limitations

The study has some limitations that should be taken into account. First, the small sample size (36 participants; 17 and 19 per group) limits the statistical power and increases the risk of type II errors, baseline imbalances, and the overestimation of effect sizes. Consequently, these findings should be considered preliminary and interpreted with caution, primarily serving to stimulate further research and critical reflection within the literature. Indeed, these findings must be verified in larger cohorts with a wider range of risk variables and sociodemographic backgrounds, as the results obtained in a monocentric setting may not adequately represent groups with greater levels of comorbidity. Moreover, longitudinal assessments are absent—an aspect that is crucial for understanding whether training may influence the perception of fatigue over time. Future studies with larger samples, including those conducted across multiple rehabilitation centers, are needed to confirm these findings and enhance the generalizability of the results. This approach will allow for a more precise understanding of fatigue in DN.

Another limitation concerns the assessment of fatigue. The Borg scale mainly reflects perceived exertion during physical activity and may not adequately capture chronic, cognitive, or daily life fatigue. The inclusion of multidimensional fatigue instruments could provide a more comprehensive evaluation of fatigue in future studies.

Finally, the instrumental analysis revealed no significant differences between groups. This finding may indicate that both interventions elicited comparable neuromuscular fatigue responses or that the limited sample size prevented the detection of subtle differences. Moreover, only the tibialis anterior muscle was examined, while dry needling potentially affects several peripheral nerves. This single-muscle assessment limits the comprehensiveness of the physiological interpretation. Future studies should aim to overcome these measurement gaps to provide a broader picture of neuromuscular adaptations, and the present findings should be interpreted in light of these potential sources of bias.

## 5. Conclusions

Exercise plays an essential role in DM therapy, reducing morbidity/mortality through aerobic/resistance training, as per ADA recommendations [25]. However, effects on DN functional capacity remain underexplored [67,103]. Our multimodal approach (clinical/sEMG fatigue assessment) contributes to bridging this gap, showing that both training modalities reduce fatigue, without highlighting clear superiority in efficacy [85,88] but confirming their safety [73,104]. Overall, these patterns might guide tailored early interventions.

## Figures and Tables

**Table 1:** Between-group differences at T0.

Variable	Treadmill (N = 17)	Elastic Band (N = 19)	*p*
Age	75.0 (68.8, 79.3)	76.0 (71.0, 78.8)	0.99
BMI	27.9 (26.8, 29.9)	26.4 (25.0, 28.3)	0.09
Blood glucose	114.0 (106.3, 131.5)	96.0 (80.5, 138.8)	0.16
SpO2 pre-treatment in %	96.0 (95.0, 97.0)	97.0 (96.0, 98.0)	0.10
HR pre-treatment	72.0 (68.0, 75.3)	72.0 (68.0, 76.5)	0.89
Borg CR10 pre-treatment	1.00 (0.00, 3.00)	3.00 (2.00, 3.00)	0.039
Borg CR10 post-treatment	4.00 (3.00, 6.00)	5.00 (4.00, 6.00)	0.24
6MWT	256.0 (199.3, 306.5)	204.0 (180.8, 291.3)	0.31
FIM cognitive score	30.0 (30.0, 33.3)	33.0 (31.0, 35.0)	0.015
FIM motor score	65.0 (61.0, 66.5)	58.0 (53.0, 63.0)	0.022
FIM total score	95.0 (91.0, 99.0)	90.0 (84.0, 98.0)	0.10
Slope CV 20% (×10^3^)	−1.60 (−4.25, 1.15)	−4.90 (−11.00, −1.40)	0.15
CV intercept value 20%	3.81 (3.21, 4.56)	4.29 (3.72, 4.98)	0.14
Slope CV 80% (×10^3^)	−9.50 (−12.00, −1.70)	−5.00 (−17.00, −0.50)	0.69
CV intercept value 80%	4.18 (3.39, 4.86)	4.41 (3.72, 5.37)	0.40

Note: *p*—*p* value from Mann–Whitney U-test for the comparison between values at T0 for patients in the treadmill group and patients in the elastic band group.

**Table 2:** Spearman’s correlation coefficients (r) between BMI and clinical variables at baseline (T0). *p* values are reported in parentheses.

BMI vs. Variable	Spearman’s r (*p*)
Blood glucose	0.217 (*p* = 0.20)
SpO2 pre-treatment in %	−0.151 (*p* = 0.38)
HR pre-treatment	−0.090 (*p* = 0.60)
Borg CR10 pre-treatment	−0.304 (*p* = 0.07)
Borg CR10 post-treatment	−0.240 (*p* = 0.16)
6MWT	0.390 (*p* = 0.019)
FIM cognitive score	−0.392 (*p* = 0.018)
FIM motor score	0.069 (*p* = 0.69)
FIM total score	−0.041 (*p* = 0.81)
Slope CV 20%	−0.206 (*p* = 0.23)
CV intercept value 20%	0.165 (*p* = 0.34)
Slope CV 80%	−0.240 (*p* = 0.17)
CV intercept value 80%	0.275 (*p* = 0.11)

**Table 3:** Between-group differences at T0, stratified according to disease duration (i.e., <10 years versus >10 years).

	Disease Duration < 10 yrs N = 18	Disease Duration > 10 yrs N = 18	*p*
Age	77.0 (71.0, 79.0)	74.5 (70.0, 77.0)	0.41
BMI	27.2 (26.2, 30.4)	26.5 (25.4, 28.4)	0.28
Blood sugar	108.0 (89.0, 114.0)	121.5 (95.0, 139.0)	0.33
HR pre-treatment	70.0 (68.0, 72.0)	73.5 (68.0, 77.0)	0.25
SpO2 pre-treatment in %	96.0 (96.0, 98.0)	96.0 (95.0, 98.0)	0.74
Borg CR10 pre-treatment	2.00 (0.00, 3.00)	2.50 (1.00, 3.00)	0.54
Borg CR10 post-treatment	5.50 (3.00, 6.00)	4.50 (3.00, 5.00)	0.25
6MWT	253.0 (201.0, 306.0)	218.0 (178.0, 292.0)	0.30
FIM cognitive score	30.0 (30.0, 33.0)	33.0 (30.0, 35.0)	0.15
FIM motor score	62.5 (54.0, 65.0)	60.0 (56.0, 70.0)	0.86
FIM total score	94.5 (86.0, 98.0)	94.0 (88.0, 99.0)	0.85
Slope CV 20% (×10^3^)	−3.20 (−8.50, 1.10)	−2.95 (−10.00, −0.40)	0.63
CV intercept value 20%	3.81 (3.51, 4.54)	4.10 (3.52, 5.30)	0.19
Slope CV 80% (×10^3^)	−6.00 (−14.55, −2.15)	−5.00 (−13.00, −0.50)	0.65
CV intercept value 80%	3.92 (3.57, 4.62)	4.57 (3.42, 5.37)	0.20

Note: *p*—*p* value from Mann–Whitney U-test for the comparison between values at T0 for patients with disease duration < 10 years and patients with disease duration > 10 years.

**Table 4:** Between-group differences at T0.

	Treadmill N (%)	Elastic Band N (%)	Chi-Squared	*p*
Male	11 (65%)	12 (63%)	0.0093	0.92
Disease duration < 10 years	11 (65%)	7 (37%)	2.7864	0.10
Hypertension	15 (88%)	17 (89%)	0.0139	0.91
Dyslipidemia	6 (35%)	4 (32%)	0.9071	0.34
Retinopathy	0 (0%)	1 (5%)		1 ^
Nephropathy	1 (6%)	2 (11%)		1 ^
Insulin	4 (24%)	6 (32%)		0.72 ^
Smoking	3 (18%)	3 (16%)		1 ^
Alcohol	1 (6%)	1 (5%)		1 ^

Note: *p*—*p* value from chi-squared test or Fisher’s exact test (^) for the comparison between values at T0 for patients in the treadmill group and patients in the elastic band group.

**Table 5:** Descriptive statistics of outcome variables (median (Q1, Q3)).

	Treadmill T0	Treadmill T1	Elastic Band T0	Elastic Band T1
Blood sugar	114.0 (106.3, 131.5)	100.0 (89.0, 122.0)	96.0 (80.5, 138.8)	93.0 (75.3, 108.5)
Borg CR10 pre-treatment	1.00 (0.00, 3.00)	1.00 (0.00, 2.00)	3.00 (2.00, 3.00)	1.00 (0.00, 2.00)
Borg CR10 post-treatment	4.00 (3.00, 6.00)	3.00 (2.00, 5.00)	5.00 (4.00, 6.00)	4.00 (2.00, 5.00)
6MWT	256.0 (199.3, 306.5)	318.0 (286.0, 357.0)	204.0 (180.8, 291.3)	276.0 (240.5, 316.5)
FIM cognitive score	30.00 (30.00, 33.25)	30.00 (30.00, 33.25)	33.00 (31.00, 35.00)	33.00 (31.00, 35.00)
FIM motor score	65.00 (61.00, 66.50)	76.00 (74.75, 78.00)	58.00 (53.00, 63.00)	72.00 (66.25, 77.50)
FIM total score	95.00 (91.00, 99.00)	108.00 (104.75, 109.50)	90.00 (84.00, 98.00)	103.00 (98.25, 110.75)
Slope CV 20% (×10^3^)	−1.60 (−4.25, 1.15)	−2.70 (−5.25, −0.88)	−5.00 (−11.75, −1.15)	−2.50 (−8.25, −0.82)
CV intercept value 20%	3.81 (3.21, 4.56)	4.47 (3.59, 5.16)	4.45 (3.89, 4.98)	4.10 (3.77, 4.71)
Slope CV 80% (×10^3^)	−9.50 (−12.00, −1.70)	−6.50 (−13.00, −3.55)	−6.00 (−17.50, −0.30)	−5.00 (−20.75, −3.00)
CV intercept value 80%	4.18 (3.39, 4.86)	4.59 (3.61, 5.08)	4.64 (3.75, 5.40)	4.18 (3.69, 4.59)

**Table 6:** Efficacy of the two interventions.

	Treadmill T0	Treadmill T1	Elastic Band T0	Elastic Band T1	*p* Group (F Value, Effect Size)	*p* Time (F Value, Effect Size)	*p* Group × Time (F Value, Effect Size)
Blood sugar	114.5 (106.7, 123.0)	106.1 (98.8, 113.9)	109.2 (102.1, 116.8)	99.5 (93.0, 106.4)	0.11 (2.68, 0.07)	0.017 (6.25, 0.16)	0.81 (0.06, 0.002)
Borg CR10 pre-treatment	1.36 (0.90, 2.06)	0.79 (0.46, 1.34)	1.83 (1.33, 2.52)	0.86 (0.55, 1.34)	0.35 (0.90, 0.026)	0.003 (10.35, 0.23)	0.60 (0.28, 0.008)
Borg CR10 post-treatment	4.33 (3.42, 5.48)	3.29 (2.51, 4.31)	4.56 (3.69, 5.64)	3.22 (2.50, 4.14)	0.90 (0.02, 5.9 × 10^−4^)	0.01 (7.01, 0.17)	0.75 (0.10, 0.003)
6MWT	233.1 (221.2, 245.6)	296.7 (281.6, 312.6)	227.6 (216.7, 239.1)	275.3 (262.0, 289.3)	0.10 (3.69, 0.097)	<0.0001 (72.1, 0.68)	0.21 (0.32, 0.009)
FIM cognitive score	32.0 (31.4, 32.5)	31.9 (31.4, 32.4)	32.1 (31.6, 32.6)	31.7 (31.2, 32.2)	0.84 (0.04, 0.011)	0.33 (0.98, 0.028)	0.44 (0.60, 0.017)
FIM motor score	61.8 (60.1, 63.6)	74.1 (72.0, 76.3)	60.3 (58.7, 62.0)	73.1 (71.2, 75.1)	0.18 (1.87, 0.05)	<0.0001 (189.0, 0.85)	0.69 (0.17, 0.005)
FIM total score	93.6 (91.8, 95.4)	105.9 (103.9, 107.9)	92.5 (90.9, 94.2)	105.1 (103.2, 107.0)	0.32 (1.02, 0.029)	<0.0001 (188.25, 0.85)	0.85 (0.04, 0.001)
Slope CV 20% (×10^3^)	−3.60 (−7.00, −0.20)	−6.22 (−9.52, −2.92)	−6.26 (−9.60, −2.92)	−5.31 (−8.73, −1.89)	0.62 (0.25, 0.007)	0.26 (1.34, 0.038)	0.80 (0.06, 0.002)
CV intercept value 20%	3.99 (3.71, 4.30)	4.62 (4.28, 4.98)	4.14 (3.85, 4.45)	3.93 (3.64, 4.23)	0.10 (2.85, 0.077)	0.21 (1.66, 0.046)	0.01 (7.51, 0.18)
Slope CV 80% (×10^3^)	−7.87 (−16.07, 0.32)	−4.75 (−12.94, 3.44)	−8.14 (−15.86, −0.41)	−12.10 (−20.05, −4.15)	0.34 (0.94, 0.027)	0.92 (0.01, 2.9 × 10^−4^)	0.37 (0.81, 0.023)
CV intercept value 80%	4.13 (3.76, 4.55)	4.63 (4.20, 5.10)	4.26 (3.89, 4.67)	4.07 (3.71, 4.47)	0.31 (1.09, 0.031)	0.47 (0.54, 0.016)	0.09 (3.03, 0.082)

Note: Adjusted means (95% confidence intervals) estimated from generalized linear mixed models (GLMM) and back-transformed to the original scale. For each outcome, type III tests of fixed effects are reported with numerator and denominator degrees of freedom (df = 1,34), F statistics, corresponding *p* values, and the partial eta-squared (η^2^p) as a measure of the effect size. The partial eta-squared was calculated from the F statistics and reflects the proportion of explainable variance attributable to each predictor relative to the residual variance after accounting for other effects in the model. *p* values refer to the main effects of group and time and to the group × time interaction.

## Data Availability

The datasets generated and analyzed during the current study are available from the authors upon reasonable request due to patient privacy considerations.

## Abbreviations

The following abbreviations are used in this manuscript:

DM	Diabetes Mellitus
T2DM	Type 2 Diabetes Mellitus
DN	Diabetic Neuropathy
BMI	Body Mass Index
SpO2	Oxygen Saturation
HR	Heart Rate
6MWT	Six-Minute Walking Test
FIM	Functional Independence Measure
Borg CR10	Borg Category Ratio Scale 0–10
MNSI	Michigan Neuropathy Screening Instrument
MCV	Maximal Voluntary Contraction
CV	Conduction Velocity
sEMG	Surface Electromyography
